# Supercritical CO_2_ Extraction of Triterpenoids from Chaga Sterile Conk of *Inonotus obliquus*

**DOI:** 10.3390/molecules27061880

**Published:** 2022-03-14

**Authors:** Nghia Huynh, Gabriele Beltrame, Marko Tarvainen, Jukka-Pekka Suomela, Baoru Yang

**Affiliations:** Food Chemistry and Food Development, Department of Biochemistry, University of Turku, Itäinen Pitkäkatu 4, 20520 Turku, Finland; nghia.huynh@fi.nestle.com (N.H.); gabbel@utu.fi (G.B.); marko.tarvainen@utu.fi (M.T.); jusuom@utu.fi (J.-P.S.)

**Keywords:** *Inonotus obliquus*, SFE, supercritical fluids, triterpenoids, inotodiol

## Abstract

Triterpenoids are among the bioactive components of Chaga, the sterile conk of the medicinal fungus *Inonotus obliquus*. Supercritical fluid extraction of Chaga triterpenoids was carried out with supercritical CO_2_, while a modified Folch method was used as a comparison. Three temperature-pressure combinations were tested varying between 314–324 K (40–50 °C) and 281–350 bars, using time- and volume-limited extractions. Six triterpenoids were identified with GC-MS and quantified with GC-FID: ergosterol, lanosterol, β-sitosterol, stigmastanol, betulin, and inotodiol. The Folch extraction resulted in recovery of trametenolic acid, which was not extracted by supercritical CO_2_. Inotodiol was the major triterpenoid of all the extracts, with a yield of 87–101 mg/100 g and 139 mg/100 g, for SFEs and the Folch method, respectively. The contents of other major triterpenoids, lanosterol and ergosterol, varied in the ranges 59–63 mg/100 g and 17–18 mg/100 g by SFE, respectively. With the Folch method, the yields were 81 mg/100 g and 40 mg/100 g, respectively. The highest recovery of triterpenoids with SFE in relation to Folch was 56% and it was obtained at 324 K (50 °C) and 350 bar, regardless of extraction time or volume of CO_2_. The recoveries of lanosterol and stigmastanol were unaffected by SFE conditions. Despite the lower yield, SFE showed several advantages including shorter extraction time and less impact on the environment. This work could be a starting point for further studies on green extraction methods of bioactive triterpenoids from Chaga.

## 1. Introduction

*Inonotus obliquus* (Fr.) Pilát, is a basidiomycete, which grows mainly above the 40th parallel north in Europe, Asia and America. It is an obligate parasite of the birch tree, where it causes the protrusion of a coal-black, cracked-shape structure from the bark of branches and stem, called sterile conk. This conk, commonly called Chaga, consisting of mycelium and birch wood, is considered a folk medicine in Baltic countries, eastern Europe, Siberia and China [1]. Traditionally, Chaga extracts have been used for treating cancer and for stomach and liver diseases [2]. The studies on Chaga extracts have shown that components responsible for their biological activities, such as anti-tumor, immunomodulative, and antioxidant, are polysaccharides, phenolic compounds, and triterpenoids [3]. Among the latter, inotodiol, a lanostane triterpenoid found only in Chaga, has been the subject of extensive research as a potential anti-cancer compound. Inotodiol has been found to induce apoptosis of human cervical cancer [4], mouse leukemia [5] and human lung cancer [6] cell lines. The application of inotodiol to mice skin reduced the number of papilloma-bearer mice and the amount of papillomas per mice [7]. However, the investigation of triterpenoids extracted from Chaga showed that also other compounds of this class have anti-tumor activities [8]. For example, betulin has shown to hamper cell metabolism of hepatocellular carcinoma, and ergosterol (most commonly found in mushrooms and fungi) has shown the capacity to inhibit the growth of different tumor cell lines [9].

In most of the researches, triterpenoids are extracted and fractionated from biomasses utilizing hazardous solvents, such as petroleum ether, dichloromethane, or methanol. There is a clear need for green technologies to extract triterpenoids for application as nutraceuticals and pharmaceutical ingredients, due to the increasing evidence on the bioactivities of these compounds and the concern about the environmental impact of use and disposal of organic solvents. Moreover, this is a general issue for terpenic compounds [10,11]. Sustainable processing methods of plant materials have been a topic of growing interest in recent years. Supercritical fluid extraction (SFE), utilizing relatively non-hazardous solvents in their supercritical state, has been already widely applied for the extraction of different components from plant material [12]. In particular, the high selectivity, inert nature, low toxicity and tunable properties of supercritical carbon dioxide (SC-CO_2_) allow the extraction of target compounds from the sample material without affecting its structure or producing solvent waste. Triterpenoids are among the components which can be extracted with SC-CO_2_ from various sample matrix. They have been extracted from edible plants [13], medicinal plants [14], mushrooms [15], and wood [16].

The present work focused on the extraction of triterpenoids from the sterile conk of *I. obliquus* with pure SC-CO_2_. To the best of our knowledge, this is the first study on the extraction of bioactive components of Chaga utilizing supercritical carbon dioxide. Very recently, subcritical water was applied for the extraction of phenolic compounds, vitamins, and minerals from Chaga [17]. In this work, triterpenoids were extracted with different experimental conditions, varying in extraction time and volume of SC-CO_2_. For comparison, a modified Folch method was utilized to exhaustively extract triterpenoids from Chaga. The comparison between the results of the supercritical fluid extraction and Folch extraction provided an insight on the extraction efficiency of SFE and the potential for SFE to eventually replace the traditional methods. The triterpenoids were analyzed by gas chromatography (GC), using flame ionization (FID) and mass spectrometric (MS) detection. The compounds were identified according to their retention times (ergosterol, lanosterol, and betulin) and measured mass spectra (inotodiol, tramentenolic acid, stigmastanol, and β-sitosterol) by comparison with spectral libraries and available reference standards.

## 2. Results and Discussion

### 2.1. Extraction Yield

The triterpenoids of the sterile conk of *I. obliquus* were successfully extracted with SC-CO_2_ and quantified with GC-FID after saponification of the extracts. The details of the different extraction conditions and resulting yields and total amounts of identified triterpenoids extracted are summarized in Table 1. The highest total triterpenoid amount by SFE was obtained by using 324 K (50 °C) and 350 bar (CO_2_ density of 899.40 kgm^−3^), without significant differences between extractions performed with extraction time (15 min) or volume (50 mL) as limiting factors. With the same temperature and pressure, increasing the solvent/sample ratio from 22.2 to 31.8 (*w*/*w*) did not result in an increase in total extraction yield of triterpenoids. In industrial applications, a longer extraction time means higher costs. Therefore, the optimal extraction time and solvent/sample ratio is a compromise between the yield and the costs. On the other hand, the lowest yield of triterpenoids was obtained under two with different CO_2_ densities, 934.90 kgm^−3^ and 898.45 kgm^−3^, again independent of the limiting factor of the extraction. This suggests a lack of positive correlation between fluid density and extraction yield. From our results it could be speculated that the increase in temperature, in a single extraction apparatus, affected the yield of desired compounds more than an increase in SC-CO_2_ density. This could be attributed to the positive effect of temperature on solubility and diffusivity of triterpenoids in SC-CO_2_, as it was shown for ursolic acid [12]. However, further experiments with different pressure–temperature combinations would be required to verify our hypothesis.

Chaga is a mixture of mycelium and wood and its exact nature is only partially understood [1]. Therefore, a comparison with previous reports would be only indicative. The triterpenoid content in the bark of birch species usually hosting *I. obliquus* is higher than 3.5 *w*/*w*% [19], while the content of sterols and other unsaponifiable compounds in birch wood is 0.5 *w*/*w*% [20]. The SC-CO_2_ extraction of birch bark had a yield of 6%, using 100 times the amount of CO_2_ used in our experiments [18]. To the best of our knowledge, a SC-CO_2_ extraction of birch wood has never been performed so far. 

Domingues and co-workers extracted triterpenoids by SFE from *Eucalyptus globulus* bark by applying 40 °C with different pressure combinations between 100 to 220 bar. Without using cosolvent, the highest yield they obtained was by using 314 K (40 °C) and a 220 bar (858.7 kgm^−3^), resulting in a 73% higher total triterpenoid quantity compared to our work. However, the extraction time was twelve times longer and solvent/sample ratio was also significantly higher, at 75 (*w*/*w*). [14]. Gil-Ramírez and co-workers extracted triterpenoids from *Agaricus bisporus* fruiting bodies by SFE at 314 K (40 °C) degrees and tested several pressure conditions (90, 180 and 300 bar). Again, the solvent/sample ratio was significantly higher (128 *w*/*w*) compared to our work, as Gil-Ramírez and coworkers used 3 h per extraction. They obtained a total triterpenoid content on average 130% higher compared to the total triterpenoids content of our work. Surprisingly, the differences between the extraction yields resulting from different extraction pressures were not significant (no cosolvent) [13].

### 2.2. Identification of Triterpenoids in SFE and Folch Extracts by Mass Spectra of GC-MS

The GC-MS fragmentation patterns allowed the identification of seven triterpenoids in the SFE and Folch extracts. The identification was based on matching the recorded retention indices and fragmentation patterns with those reported in the literature (Table 2).

**Figure 1:**
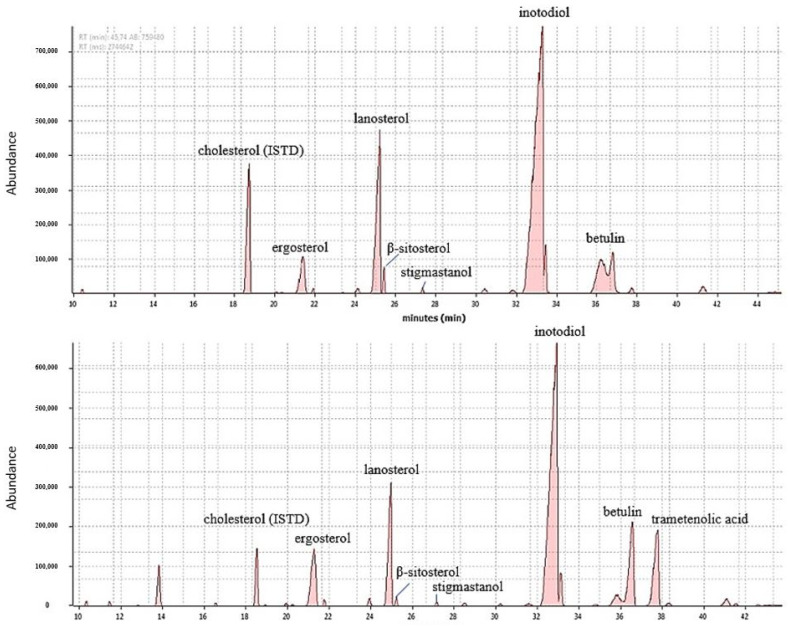
Comparison between chromatograms of GC-MS (total ion chromatogram) obtained by SFE (**upper**) and Folch method (**lower**).

Examples of chromatograms are reported in Figure 1. Lanosterol was identified based on its fragmentation pattern with the molecular ion (M^+^) at *m/z* 498 and ions at *m/z* 131 [TMSiO-CH_3_] and *m/z* 337 [M^+^—131]. Additionally, the fragmentation ions at *m/z* 255 and 229 were previously identified as typical for Δ^8^ -sterols [23]. The mass spectrum of the strongest peak matched well the spectrum of the TMS ether of inotodiol published in the literature and was identified as lanosta-8,24-diene-3β,22R-diol. Our observed fragmentation ions for inotodiol were in agreement with results by Kahlos et al., with the only difference being that the molecular ion M^+^ was not present. The intensity of the molecular ion in the work of Kahlos et al. was also a low, at 7% [21,22]. Trametenolic acid was identified based on its fragmentation pattern with the molecular ion at *m/z* 600 and relatively intense ions at *m/z* 281, *m/z* 187, *m/z* 585, which matched with observations made previously by Kahlos et al. [21,22]. The mass spectra of inotodiol and trametenolic acid also showed an abundant ion at *m/z* 69, which is typical for the fragmentation of the Δ^24(25)^ -side chain [23]. Ergosterol showed a base peak at *m/z* 363, resulting from the breakdown of TMSiOH and a methyl group from the C-ring [24]. Ergosterol mass spectra also showed a molecular ion at *m/z* 468, matching the literature reference. The cholesterol (3β-cholest-5-en-3-ol) TMS ether showed the molecular ion at *m/z* 458 with a low relative intensity, and a similar observation was made by Kahlos et al. in their work. Cholesterol mass spectra showed relatively intense peaks at *m/z* 353 [M^+^—TMS—CH_3_] and *m/z* 255 [M^+^—SC—ROH—CH_3_], owing to the cleavage of the trimethylsilyl-, methyl-, hydroxyl group and the side chain [23,25]. The observed ion in the mass spectra of cholesterol at *m/z* 368 results from the fragmentation mechanism of free Δ^5^-sterols, including losses of carbons from the sterol A- and B–rings by the retro-Diels–Alder reaction [26]. 

The β-sitosterol and stigmastanol quantities were the lowest, which also showed as lesser fragments making the identifying by GC-MS more challenging, especially in the case of stigmastanol. The base peak for β-sitosterol derives from the loss of the TMSi-group with C1, C2 and C3 in the sterol A-ring. The second most abundant ion at *m/z* 357 derived from [M^+^—129] with 72% abundancy, and the third most abundant ion was due to the loss of TMSiO. Both cholesterol and β-sitosterol showed an intense ion at *m/z* 129, which is characteristic for silylated Δ^5^–sterols [25]. The identification of stigmastanol was also done by comparing the retention time and mass spectra with those of the TMS ethers of the reference compounds. The mass spectra of betulin exhibit an intense peak at *m/z* 189, which is characteristic for the fragmentation of triterpenoid molecules with a lupane skeleton with a hydroxy group in position 3. Betulin, lupeol, betulinic acid and lupenone are considered as representative of triterpenes with a lupane structure [27]. This ion arises from the fragmentation of the C ring system by cleavage of the C9–C11 and C8–C14 bonds followed by the loss of an H_2_O molecule. Another abundant fragment ion is at *m/z* 203, which is related to the retention of an additional methylene group from the C ring with respect to the fragment ion at *m/z* 189 [28,29]. The presence of betulin was also confirmed by the comparison of retention time against the betulin standard.

### 2.3. Quantification of Triterpenoids in SFE and Folch Extracts

The major triterpenoids in the SFE extract were, in the order of abundance, inotodiol, lanosterol, ergosterol, betulin, β-sitosterol, and stigmastanol. In addition to these compounds, the Folch extracts contained also trametenolic acid. It was the most abundant compound after inotodiol and lanosterol. The amount of individual compounds extracted with SC-CO_2_ and the Folch method from Chaga, expressed as mg/100 g of Chaga, are reported in Table 3. The main triterpenoid extracted from *I. obliquus* was inotodiol, in the range 87–101 mg/100 g for SFE and 139 mg/100 g with Folch. The second and third most abundant triterpenoids were lanosterol and ergosterol, the amount extracted with SFE being 59–62 mg/100 g and 17–18 mg/100 g, respectively, and of 81 mg/100 g and 41 mg/100 g, respectively, extracted by the Folch method. The total amounts of triterpenoids extracted from Chaga in the current study (180–350 mg/100 g Chaga) were more similar to the total amount extracted from birch heartwood with petroleum ether (259 mg/100 g), but remarkably lower than the total amount obtained with methanol from *I. obliquus* mycelium (about 970 mg/100 g), as reported by Wang and co-workers [30]. The discrepancy could be attributed to a larger extent to the starting material, rather than the extraction method, since the Folch solvent had a δ of 25.53 MPa^1/2^, equidistant between methanol (36 MPa^1/2^) and petroleum ether (14.9 < δ < 16.8 MPa^1/2^).

**Figure 2:**
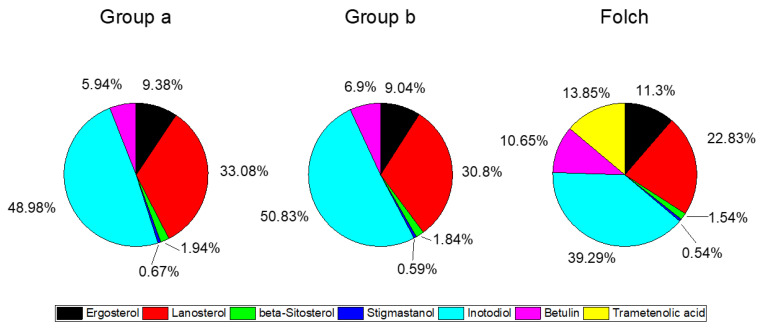
Relative compositional profile of the obtained triterpenoids of SFE and Folch extracts. SFE extracts are averaged and grouped according to the compact letter display of the total triterpenoid (mg/100 g Chaga) amounts reported in Table 1.

Complex fractions with different compositions would have different biological activities, therefore, the relative composition in triterpenoids should be taken into account when selecting and optimizing extraction methods. As can be observed in Figure 2, the relative composition of triterpenoids extracted by SFE and the Folch method was relatively similar. The Folch extract showed high amounts in inotodiol, which comprised nearly 40% of the total triterpenoid content. The relative amount of inotodiol increased to 50% in the SC-CO_2_ extraction, carried with a fluid density of 899.40 kgm^−3^. In the Folch extract the lanosterol was followed in abundance by trametenolic acid (49 mg/100 g, 13.85% of total triterpenoids), a compound which was absent from SC-CO_2_ extracts. Ergosterol was then the fourth triterpenoid in abundance in the Folch extract. In all the extracts, β-sitosterol and stigmastanol were found in lower quantities compared to the other triterpenoids, comprising less than 2% of the total triterpenoid contents. Betulin constituted on average 5.9–6.9% and 10.7% of total triterpenoids extracted by SFE and the Folch method, respectively, in sharp contrast with the SC-CO_2_ extracts of birch bark, in which betulin constituted 63% of the total triterpenoids. Moreover, the relative amount of betulin increased to 80% in the reference n-heptane extract from birch bark [31]. The relative amounts of triterpenoids in the ethanol extract of Chaga were reported by Zheng and coworkers [32], which were different from the composition of the extracts obtained with SC-CO_2_ and the Folch method in our study. In their study, the ethanol extract of wild Chaga contained mainly lanosterol (45.5% of the total sterols), inotodiol (25.4%) and another ten sterols (30.2%), including trametenolic acid (13.9%) and intermediates in the pathway of ergosterol biosynthesis [32]. Inotodiol is a triterpenoid compound unique for Chaga, which is considered one of the major compounds responsible for the biological activities of Chaga. In our study, inotodiol accounted for about 50% of the total triterpenoids extracted from Chaga using SC-CO_2_, significantly higher than the relative abundance in the ethanol extract. 

Wang and co-workers have reported mycelial contents of ergosterol, sitosterol, and lanosterol of about 85 mg/100 g, 130 mg/100 g, and 104 mg/100 g, respectively (re-elaborated data of [30]), which were 2, 26, and 1.3 times, respectively, of the content found in Chaga in our study. In a recently published work, triterpenoid fractions were produced from Chaga with 80% methanol. The triterpenoid contents of bark and inner layers of Chaga were reported as 432 mg/100 g and 251 mg/100 g, respectively, which were comparable with our results, taking into account the fact that our samples were produced from the whole Chaga without pre-fractionation. In their work, authors have reported content of inotodiol of 151.4 g/100 g and 78.5 g/100 g for bark and inner layers, respectively [33]. These values were lower than the triterpenoid concentrations obtained using SC-CO_2_. Moreover, the authors used an extraction time of 24 h, much longer than the time used in SFE in our study (Section 3.3). These observations highlight the advantages of the utilization of SC-CO_2_ for the production of triterpenoid fractions.

### 2.4. Recovery of Triterpenoids with SFE

As mentioned above, both the total triterpenoid content and amount of individual triterpenoid compounds obtained with the Folch method were significantly higher than those obtained with SFE. Folch is considered the protocol of choice for the exhaustive extraction of lipids. However, compared to SFE, the solvent/sample mass ratio required was more than double (Table 1). More importantly, Folch uses a mixture of dichloromethane and methanol, therefore creating bulk hazardous waste. Therefore, we estimated the relative amount of triterpenoids extracted by SC-CO_2_ in comparison with the traditional Folch method. The results of comparison are reported in Table 3. The compound with the highest recovery was lanosterol (75% on average), regardless of the different extraction conditions, while the triterpenoid with the lowest recovery was betulin (26% after time-limited extraction with a CO_2_ density of 898.45 kgm^−3^). The recovery of inotodiol was significantly influenced by the extraction conditions, and the highest recoveries (72–73%) were obtained with a CO_2_ density of 899.40 kgm^−3^, regardless of the extraction limited by time or SC-CO_2_ volume. Additinally, the highest recovery of betulin was obtained at these conditions, while the highest recovery of ergosterol was obtained with the same fluid density only after the extraction was limited by time (Table 4). As could be expected from these results, the highest recovery of total triterpenoids was obtained with the same conditions of inotodiol (a CO_2_ density of 899.40 kgm^−3^).

## 3. Materials and Methods

### 3.1. Chemicals

Liquid carbon dioxide (purity 0.9999) was supplied by AGA Oy (Turku, Finland). Methanol and acetonitrile (HPLC grade, purity 0.999) were purchased from WVR International. Dichloromethane (HPLC grade, purity 0.999) was purchased from Labscan, Pathumwan, Thailand. Ethanol (technical grade) was purchased from Altia Industrial, Rajamäki, Finland. Tri-Sil reagent was purchased from ThermoFisher (Waltham, MA, USA). Cholesterol (≥99%), ergosterol (≥75%), lanosterol (≥93%), and betulin (≥97.5%) were purchased from Sigma-Aldrich (St. Louis, MO, USA).

### 3.2. Sample Material

Coarse ground and commercially available wild Chaga (sterile conk of *I. obliquus*) was supplied by Eevia Oy (Seinäjoki, Finland). The moisture content was 8% (*w*/*w*), and particle sizes varied from 10 to 20 mm. Chaga was crushed by using a mortar and pestle, and a distribution of particle sizes around 1 mm in diameter was selected after centrifugal milling (Retsch ZM1, Retsch, Haan, Germany). The same batch was utilized throughout the work. It was stored in screwcap 250 mL glass bottles at room temperature, away from direct sunlight. Work was carried within three days after the sample milling. Prior to extraction, the samples were weighed and placed in 10 mL plastic extraction cartridges along with an inert filling material (Leco Dry, Leco Corporation, St. Joseph, MO, USA). The porous filling material was used to prevent dead volume inside the cartridge. The filling material was supplied by the equipment manufacturer.

### 3.3. SFE Apparatus and Procedure

The SC-CO_2_ extractions of the present work were performed with an Isco supercritical fluid extractor (model Isco SFX 200, ALT, East Lyme, CT, USA). The integrated system consists of two Isco model 260D pressurizing syringe pumps, an Isco SFX 200 control panel, a temperature-controlled reactor chamber with two ports (i.e., a heating extraction chamber), a pressure reducing restrictor capillary (i.e., the restrictor for short) and a stand for collection tubes. A 70 cm long capillary restrictor tube with an inner diameter of 70 µm was applied. Three hundred nanoliters of the internal standard solution (1 g/mL free cholesterol in hexane) were added directly into the samples (2 g) in the extraction cartridge. The extractions were carried out as off-line extractions with dynamic extraction mode.

The extraction system was cleaned between the extractions by performing a 10 min extraction with a blank cartridge. The adequacy of the cleaning was verified by doing two blank extractions, of which the second extract was analyzed to confirm the absence of any triterpenoid residues. The 10 mL plastic extraction cartridges were washed with soap, dried and then sonicated in methanol. Prior to use, the cartridges were always rinsed with ethanol and dried.

The extraction time of the apparatus could be limited by time or fluid volume. Both conditions were tested. The flow velocity of the extraction was mainly controlled by the outlet capillary tubing, which is part of the restrictor system at the end of the extraction cycle. A 1.0 m long, fused silica capillary (100 μm I.D., 363 μm O.D.) was used, and this resulted in approximately 3 mL/min flowrate. The temperature of the restrictor was kept at 329 K (55 °C) to prevent clogging of the capillary. The chosen fluid volume (50 mL) was equal to five times the internal volume of the sample cartridge. The average extraction time of volume-limited extraction was 13 ± 0.47 min, which is comparable to the time-limited extractions. Extractions were performed in triplicate.

### 3.4. Folch Extraction

The Folch extraction procedure was modified from the original method presented by Folch and co-workers [34]. An aliquot of 300 μL of the internal standard solution (1 g/mL in hexane) was added into the Chaga sample (2 g) prior to extraction. The sample was homogenized for three minutes with 60 mL of dichloromethane:methanol (2:1, *v*/*v*) mixture. The sample was then filtered with 0.45 μm filter paper, and solid material was scraped back to a beaker. The extraction steps were repeated three times in total. The extracts of the three extractions were combined, transferred to a separation funnel with 120 mL of diethyl ether, and washed with saturated NaCl solution. After thorough mixing, the mixture was allowed to separate, and the upper phase was completely recovered. The second washing step was carried out with purified water. After thorough mixing, the water phase was carefully discarded and the lipids containing solvent phase completely recovered. Finally, the solvents were removed with rotary evaporator. The Folch extraction was performed in triplicate. 

### 3.5. Saponification of the Extracts

The Folch and SC-CO_2_ extracts were saponified to remove triacylglycerols and other free fatty acids from the extracts. To each extract, 20 mL of 1.2 M KOH in ethanol was added, and the mixture was then heated with mixing at 344 K (70 °C) for 60 min. To reduce oxidation of sterol and triterpenoids, the saponification vessel was purged with nitrogen prior to heating, and then sealed with parafilm and aluminum foil to reduce the presence of oxygen during saponification. After hydrolysis, 40 mL of saturated NaCl solution and 120 mL of diethyl ether were added to the flask, followed by shaking. The mixture was then separated in a separatory funnel. The ether phase was collected by discarding the aqueous phase, and washed with 40 mL of water. The aqueous phase was again discarded, and the remaining ether phase was evaporated and dried with N_2_ flow.

### 3.6. Gas Chromatographic Analysis

The samples and standards were initially pipetted into 2.5 mL vials and held under nitrogen flow to evaporate the solvent. After drying, the Tri-Sil reagent was added for the derivatization, which was carried at 70 °C for 1 h. The samples were quantitatively transferred into 300 μL inserts, which were placed inside 2.5 mL sample vials. Identification of triterpenoids was performed by GC-MS (model HP 6890, HP Inc., Palo Alto, CA, USA) equipped with a DB5 30 m × 0.25 mm × 0.25 μm column (Agilent Technologies, Santa Clara, CA, USA). The mass spectrometer was operated in positive ion mode with ionization energy of 70 eV. Helium was used as the carrier gas at a flow rate of 1 mL/min and linear velocity of 30 cm/s. 

The column temperature was initially set at 255 °C (held for 1 min), then increased to 265 °C at 0.75 °C/min, held at 265 °C for 25 min, increased to 325 °C at 10 °C/min and finally held at 325 °C for 5 min. A gas chromatograph (model GC-2010 plus, Shimadzu, Kyoto, Japan) equipped with a flame ionization detector and a DB1-MS 30 m × 0.25 mm × 0.25 μm column (Agilent Technologies, Santa Clara, CA, USA), was used in the quantitative analysis of the triterpenoids. The samples were analyzed in triplicates. An equivalent oven temperature program was used as in the GC-MS analysis. Quantification was performed using cholesterol as internal standard. Calibration curves of betulin, ergosterol, and lanosterol were obtained and used for quantification of these compounds. In addition, the calibration curve of betulin was used for the quantification of inotodiol, and the calibration curve of ergosterol was used for the quantification of trametenolic acid, β-sitosterol and stigmastanol. 

### 3.7. Statistical Analysis

The statistical analysis was performed with RStudio [35]. Normality of the data was assessed with Shapiro–Wilk test and Levene test was used for assessing the homogeneity of data variance. Packages *dlookr* and *car* were used. The analysis of variance was performed with the functions *aov* and *TukeyHSD* (*stats* package). Differences reaching a confidence level of 95% (*p* < 0.05) were considered statistically significant.

## 4. Conclusions

Triterpenoids were successfully extracted from Chaga utilizing pure SC-CO_2_. The total yield of triterpenoids obtained by the Folch method was higher compared to SFE (*p* < 0.05), which was expected due to the exhaustiveness of the Folch procedure. In addition, trametenolic acid was found only in the Folch extract, likely due to the higher polarity of the solvents, compared to SC-CO_2_. However, compared to the Folch method, SFE had a shorter extraction time and did not require additional cleaning or solvent removal steps. Among the extraction parameters studied, the highest recoveries of triterpenoids were obtained with SC-CO_2_ at 324 K (50 °C) and a 350 bar; in particular, inotodiol, one of the most investigated bioactive compounds of Chaga, was extracted with a high recovery rate (72% relatively to the using the Folch method) using these temperature and pressure parameters. This study demonstrated the feasibility of supercritical fluid CO_2_ extraction as a green technology for extracting triterpenoids from Chaga. In this study, we used temperature and pressure parameters that have been often used for extracting sterols with supercritical CO_2_. Using pressures higher than a 350 bar may further increase the yield, as well as the coextraction of other lipids. Further research is worth to perform in order to investigate the impact of, e.g., higher extraction pressures on the yield and composition of the extracts.

## Figures and Tables

**Table 1:** Extraction yield and total extracted terpenes using SFE and Folch methods and corresponding extraction conditions. Data are expressed as means ± standard deviation. The different letters (a–c) indicate significant difference (*p* < 0.05).

Extraction Limiting Factor	Temperature (K)	Temperature (°C)	Pressure (bar)	CO_2_ Density (kgm^−3^)	Hildebrand Solubility Parameter * (MPa^1/2^)	Solvent/ Sample (g/g)	Extraction Yield ^#^ (%)	Total Triterpenoids (mg/100 g Chaga)
50 mL	314	40	281	898.45	15.95	22.22 ± 0.16	0.06 ± 0.02 ^a^	184.85 ± 6.05 ^a^
50 mL	324	50	350	899.40	15.96	22.42 ± 0.06	0.11 ± 0.02 ^b^	197.66 ± 15.08 ^b^
50 mL	314	40	350	934.90	16.59	23.17 ± 0.13	0.07 ± 0.03 ^a^	179.66 ± 3.81 ^a^
15 min	314	40	281	898.45	15.95	22.21 ± 1.49	0.14 ± 0.07 ^b^	179.60 ± 5.18 ^a^
20 min	324	50	350	899.40	15.96	31.83 ± 2.04	0.06 ± 0.02 ^a^	197.21 ± 8.67 ^b^
Folch	-	-	-	-	25.53	102.36 ± 0.52 ^§^	1.21 ± 0.01 ^c^	352.73 ± 4.04 ^c^

* calculated with data from [12] and tabulated Hildebrand parameters [18]; ^#^ raw extracts; ^§^ value based on three Folch extractions, not taking into account washing steps.

**Table 2:** GC-MS spectrometric data of the TMS ether of the main triterpenoids.

Triterpenoid	Reference ^b^		Fragment Ions (*m/z*) ^a^					
Lanosterol	1	393 (100)	69(78)	498 (52, M^+^)	483(40)	109 (32)	187 (12)	227 (11)
Inotodiol	2	393 (100)	69 (98)	498 (31, M^+^)	483 (21)	109 (38)	187 (16)	227 (14)
	1	297 (85)	517 (73)	571 (65)	427 (57)	387 (33)	337 (10)	586 (7, M^+^)
Trametenolic acid	2	297 (85)	517 (31)	571 (5)	427 (17)	387 (5)	337 (100)	-
	1	281 (24)	187 (24)	213 (17)	585 (7)	495 (7)	405 (3)	600 (3, M^+^)
Ergosterol	2	281 (31)	187 (21)	213 (9)	585 (31)	495 (19)	-	600 (13, M^+^)
	1	363 (100)	69 (85)	337 (58)	468 (56, M^+^)	253 (55)	131 (48)	378 (28)
Cholesterol	2	363 (100)	69 (48)	337 (65)	468 (19, M^+^)	-	131 (20)	378 (19)
	1	129 (100)	229 (44)	107 (40)	121 (36)	368 (25)	353 (15)	255 (15)
β-sitosterol	2	129 (100)	-	107 (29)	-	368 (71)	353 (33)	255 (17)
	2	129 (100)	357 (72)	396 (67)	73 (51)	75 (33)	-	-
Stigmastanol	2	69 (100)	393 (46)	73 (40)	75 (25)	-	-	-

^a^ relative ion current intensity in brackets; ^b^ literature references [21,22] (1) our experimental results (2).

**Table 3:** The amount of the identified triterpenoids extracted from Chaga with SC-CO_2_ and Folch method, expressed as mg/100 g dry weight of Chaga. Data are expressed as mean ± standard deviation. The different letters (a–c) indicate significant difference (*p* < 0.05). (*n* = 9, 3 extraction replicates and 3 analytical replicates).

Extraction Limiting Factor	Temperature (K)	Pressure (bar)	CO_2_ Density (kgm^−3^)	Ergosterol (mg/100 g Chaga)	Lanosterol (mg/100 g Chaga)	β-sitosterol (mg/100 g Chaga)	Stigmastanol (mg/100 g Chaga)	Inotodiol (mg/100 g Chaga)	Betulin (mg/100 g Chaga)	Trametenolic Acid (mg/100 g Chaga)
50 mL	314	281	898.45	16.74 ± 0.7 ^a^	59.87 ± 5.05 ^a^	3.47 ± 0.23 ^a,b^	1.33 ± 0.09 ^a^	91.34 ± 7.37 ^a^	12.11 ± 0.82 ^a^	n.d.
50 mL	324	350	899.4	17.19 ± 0.54 ^a^	62.65 ± 4.26 ^a^	3.69 ± 0.31 ^b^	1.29 ± 0.5 ^a^	99.63 ± 6.29 ^b^	13.21 ± 5.48 ^a^	n.d.
50 mL	314	350	934.9	17.05 ± 0.57 ^a^	58.83 ± 2.08 ^a^	3.19 ± 0.23 ^a^	1.12 ± 0.43 ^a^	87.69 ± 1.07 ^a^	11.80 ± 0.84 ^a^	n.d.
15 min	314	281	898.45	17.27 ± 0.48 ^a^	61.33 ± 2.49 ^a^	3.91 ± 0.6 ^b^	1.23 ± 0.47 ^a^	87.48 ± 5.83 ^a^	8.39 ± 5.31 ^a^	n.d.
20 min	324	350	899.4	18.49 ± 0.58 ^b^	58.98 ± 4.28 ^a^	3.57 ± 0.23 ^a,b^	1.04 ± 0.6 ^a^	101.08 ± 3.56 ^b^	14.05 ± 5.37 ^a^	n.d.
Folch	-	-	-	39.86 ± 1.46 ^c^	80.54 ± 0.98 ^b^	5.43 ± 0.36 ^c^	1.90 ± 0.21 ^b^	138.58 ± 4.42 ^c^	37.58 ± 3.96 ^b^	48.84 ± 0.95

**Table 4:** Proportion (*w*/*w*%) of single compounds and total triterpenoids obtained with SFE in relation to the amount obtained with Folch extraction. The different letters (a–c) indicate significant difference (*p* < 0.05).

Extraction Limiting Factor	Temperature (K)	Pressure (bar)	CO_2_ Density (kgm^−3^)	Ergosterol (%)	Lanosterol (%)	β-Sitosterol (%)	Stigmastanol (%)	Inotodiol (%)	Betulin (%)	Total Terpenes (%)
50 mL	314	281	898.45	41.91 ± 1.04 ^a^	74.35 ± 6.84 ^a^	63.63 ± 4.02 ^a.b^	70.31 ± 9.2 ^a^	66.17 ± 5.37 ^a.b^	32.39 ± 4.97 ^a.b^	52.41 ± 1.72 ^a^
50 mL	324	350	899.40	43.11 ± 2.48 ^a^	77.72 ± 4.56 ^a^	67.66 ± 4.61 ^a^	76.15 ± 8.74 ^a^	72.16 ± 4.3 ^b.c^	39.53 ± 11.52 ^a^	56.04 ± 4.27 ^b^
50 mL	314	350	934.90	42.74 ± 2.16 ^a^	73.04 ± 3.35 ^a^	58.79 ± 7.4 ^a^	66.34 ± 10.67 ^a^	63.54 ± 2.18 ^a^	31.30 ± 2.47 ^a.b^	50.94 ± 1.08 ^a^
15 min	314	281	898.45	43.31 ± 2.22 ^a^	76.11 ± 2.82 ^a^	71.7 ± 9.79 ^b^	72.25 ± 5.74 ^a^	63.47 ± 5.8 ^a^	26.36 ± 13.37 ^b^	50.92 ± 1.47 ^a^
20 min	324	350	899.40	46.33 ± 1.31 ^b^	73.16 ± 4.69 ^a^	65.84 ± 7.03 ^a.b^	70.9 ± 12.1 ^a^	73.28 ± 4.1 ^c^	42.90 ± 8.08 ^a^	55.91 ± 2.46 ^b^

## Data Availability

Data sharing not applicable.
